# The impact of current failures on predicted well-being for future success: Different mechanisms of action in high and low self-threat situations

**DOI:** 10.3389/fpsyg.2022.954583

**Published:** 2022-12-22

**Authors:** Zhijun Hou, Yuting Wang, Lin Li, Jingjing Song

**Affiliations:** ^1^Institute of Education, China University of Geosciences, Wuhan, China; ^2^Research Center for Psychological and Health Sciences, China University of Geosciences, Wuhan, China

**Keywords:** self-threat, predicted well-being, affect, self-esteem, current performance

## Abstract

This study explored the effect of current performance on the predicted well-being for future success and its mechanism. This empirical research consists of two experiments. In Study 1, the individual’s predicted well-being of future performance in the tests was lower in good feedback condition compared with bad feedback condition. It means that individuals have a higher expectation of future success after an unimportant loss. Study 2 focused on the moderating role of self-threat situations and the mediating role of affect and self-esteem in the effect of current performance feedback on predicted well-being. The results showed that individuals who got bad feedback have a low predicted well-being of future success only in a high self-threatening condition. Self-threat plays a moderating role between current performance and predicted well-being. The serial mediation role of affect and self-esteem in the negative effect of current performance on predicted well-being holds in high self-threat situations. By specifying the behavioral consequences and analyzing the psychological process in high and low self-threat situations, this research expands the literature on development of appropriate cognitive theories and propose novel measures and practical implications of enhancing predicted well-being.

## 1. Introduction

### 1.1. The relationship between current failure and predicted well-being

The ranking of performance in a task affects the experience of well-being in people. According to social comparison theory (Festinger, 1954), when there is a lack of self-evaluation criteria, individuals tend to reduce uncertainty by comparing themselves to others who are similar. One of the sources of well-being is a comparison with others in society. Thus, people who perform well on tasks tend to feel more current well-being than those who perform poorly. Predicted well-being refers to individuals’ well-being when they envision future success in a task. The results of previous studies related to current failure and predicted well-being are divergent (Feather, 1966; Blascovich and Tomaka, 1996; Blascovich et al., 2004; Dimenichi and Richmond, 2015; Baumeister et al., 2018; Chen and Qu, 2021; Newman and Nezlek, 2022). One study confirmed that, after experiencing an initial failure, individuals tend to lower their expectations of future good performance, whereas after achieving initial success, individuals increase their expectations of future good performance (Feather, 1966). In this study, failure was discouraging and success was encouraging. However, some researchers’ experiments have shown the opposite: reflecting on failure can enhance individuals’ courage, motivation, and performance (Dimenichi and Richmond, 2015). It seems that failure is more motivating at this point.

In real life, people typically respond to failure in two beliefs. One typical belief is sour grapes. Will people mock and belittle predicted well-being for future success like the fox eating sour grapes (“belittle-the-goal” hypothesis)? The other typical belief is that some people tend to manage their expectations with such notions as “What is valuable is rare,” or “What you cannot get is better.” Therefore, these people will continue to challenge after failure and believe that they will have a higher predicted well-being for future success (“grasp-the-nettle” hypothesis). These two opposing approaches (“belittling the goal” or “grasping the nettle”) both seem to be valid. Nevertheless, they cannot be correct simultaneously under the same conditions. It’s worth exploring whether individuals are of high or low well-being after failure. Therefore, we proposed H1.

*H1*: After a current failure, individuals have higher predicted well-being of future success.

### 1.2. Moderating effect of self-threat

Bad feedback not always led to negative reaction and expectation. For example, experiments have shown that participants who are sure they have lost a game have more negative expectations and *post hoc* reconstructions than those who believe they still have a fair chance (Wilson et al., 2004). Whether people feel self-threat might influence people’ reaction and expectation after bad feedback (Qu and Lim, 2016). Some researchers have found that predicting participants’ well-being from a observer perspective and improving a poor test result to an excellent one resulted in higher levels of predicted well-being than those who consistently performed well (Sjstad et al., 2020). Another study showed similar results that showing the failure experience increase observers’ intention to try (Luan and Li, 2022). Thus, we suspected that the self-threat plays a moderation role in the relationship between bad feedback and predicted well-being.

Self-threat is defined at the operational level as an experience of failure (Hakmiller, 1966). According to this definition, negative experiences that people inevitably encounter in real life, such as poor performance, neglect in exams and jobs, or lower-than-expected results, can produce self-threat for individuals (Steele, 1988). The implicit standards of competition and comparison may predispose individuals to focus on differences in abilities between themselves and others which causes individuals to perceive the self as different from the standard, thus, triggering self-threat (Han and Chi, 2012). In addition, the biopsychosocial model defined challenge and threat as two states that occur after assessing situational needs and personal resources in a goal-related performance environment (Blascovich and Tomaka, 1996; Blascovich et al., 2004). Life experience tells us that the sense of threat from failing a vital exam is often higher than the threat from losing a game. Therefore, contexts may affect self-threat. One study found that, in situations where the outcome is attributed to ability, individuals had higher expectations of success after completing a crossword puzzle and lower expectations after the inability to complete a crossword puzzle (Mcmahan, 1973). In contrast, in situations where the outcome is attributed to luck, individuals had lower expectations of success after completing a crossword puzzle and higher expectations after failure to do so (Mcmahan, 1973). The difference between the two seems to explain the divergence between the belittle-the-goal and grasp-the-nettle scenarios that have characterized previous research findings related to current failure and predicted well-being. Therefore, using the luck-based task as a low self-threat situation, H2 is proposed.

*H2*: In a low self-threat situation, the worse the individual’s current performance (the lower ranking), the higher the predicted well-being (the grasp-the-nettle hypothesis).

Average adults are motivated to protect, maintain, or enhance their self-concept. When threatened by the ego, the individual’s initial motivation to learn is disrupted, causing the individual to ignore failure and stop processing information (Oettingen et al., 2012; Eskreis-Winkler and Fishbach, 2019, 2022). People in self-threat states are negatively affected by stress, anxiety, and other factors and perform poorly on tasks (Jamieson et al., 2018). Threat is a state of avoidance motivation, which occurs when the assessment of situational needs exceeds personal resources, and changes in threat assessment and positive affect mediate the impact of the task frame on task performance (Chen and Qu, 2021). Thus, the individual acts to counteract and minimize the threat, that is, engage in psychological defense (Sedikides, 1993). There are three main types of defenses against self-threats: compensation, breaking, and resistance (Vandellen et al., 2011; Liu et al., 2017). Compensation refers to undermining the importance of negative information when a person feels threatened and compensating for negative feedback in the moment with other strengths, such as a low score in test. Conversely, people who adopt a compensatory defense strategy emphasize their abilities in other areas (Liu et al., 2017). Breaking refers to people undermining their existing self-concept after being threatened and choosing to lower their expectations rather than deny the negative information. For example, individuals avoid disappointment by preparing for losses and protecting themselves from possible disappointment by reducing expectations or even predicting the worst-case scenario (Shepperd et al., 2000). Resistance is the refusal to acknowledge negative information about oneself when threatened. An example of resistance would be when an individual performs poorly on an ability test and declares that his or her level of ability is not related to personal identity or future success in life (Sjstad et al., 2020). In summary, after experiencing failure in the present, individuals stop processing the current event and focus on getting rid of the threat and maintaining self-esteem consequent to the ego threat. Accordingly, the belittle-the-goal hypothesis seems more likely to hold in general when experienced self-threat. Therefore, we propose H3.

*H3*: In a high self-threat situation, the worse the individual’s current performance (the lower ranking), the lower the predicted well-being (the belittle-the-goal hypothesis).

Thus, the grasp-the-nettle hypothesis is valid for the low-self-threat situation, and the belittle-the-goal hypothesis is reasonable for the high-self-threat situation. The self-threat situations may be the boundary condition that explains the frequency of either grasping the nettle or belittling the goal. This explains the moderating role of the self-threat situations. Therefore, we proposed H4.

*H4*: Situational self-threat plays a moderating role between current performance and predicted well-being.

### 1.3. The mediating role of affect and self-esteem

It is necessary to further explore the mechanism of poor current performance led to low predicted well-being in high self-threat condition. We suspected the affect and self-esteem might be important mediators. Affect may mediate the relationship between present moment performance and predicted well-being. Affect is one of the most essential components of subjective well-being (Diener, 2000). Based on Diener’s point of view, scholars have widely agreed that life satisfaction, positive affect, and negative affect are the three main components of subjective well-being. According to the cognitive theory of emotions, different people show different emotional responses to the same event or situation because people differ in their mental evaluation (attribution) of environmental events (Arnold, 1960). In other words, feedback received influences an individual’s affect. A meta-analysis found that anticipated emotions have a more substantial influence on decision-making and behavior than affect in the present (Baumeister et al., 2016). Just as people expect to remain miserable for a long time after a breakup with their partner but do not know the extent and duration of the impact moment, the effect on the predicted well-being during important real-life events is frequently overestimated (Morewedge and Buechel, 2013). Life events closely related to the individual and the future often include high self-threat situations. People who experience failure tend to experience more significant mood swings, deny the individual’s high relevance, end present performance, and defend themselves through strategies that devalue predicted well-being (Sjstad et al., 2020). In a low self-threat situation, which is relatively insignificant, the current performance of a task may not be as crucial to the individual and therefore does not result in an overestimation of the current affect and predicted well-being.

Self-esteem may play a mediating role between current performance and predicted well-being. Individuals’ self-esteem increases after experiencing success (Greenberg et al., 1992; Butler et al., 1994). However, failure always leads to losses, thus people are likely to be disappointed with failures and experience failure aversion, in which process individuals’ failure aversion negatively affected learning from failure through increasing the individuals’ perceived loss of self-esteem (Zhang et al., 2022); people tend to look away from failure and not pay attention to it to protect their self-esteem (Eskreis-Winkler and Fishbach, 2022). Above studies have confirmed the relationship between success and failure and self-esteem, which is consistent with the definition of self-threat (Hakmiller, 1966). However, the relationship between self-esteem and well-being may be more divergent than a simple positive correlation, as presented by most previous studies. Most studies suggest that self-esteem predicts the participants’ level of well-being with the outcome (Rosenberg et al., 1995; Dutton and Brown, 1997). Individuals with high and stable self-esteem tend to have higher well-being levels (Paradise and Kernis, 2002). However, some researchers have found results opposed to those of previous studies. For example, the correlation between the self-esteem and subjective well-being of people in Pakistani regions was not significant (Suhail and Chaudhry, 2004). Research on the predictive effects of implicit and explicit self-esteem on subjective well-being is divergent. Some find a more significant correlation between explicit self-esteem and subjective well-being and a lower correlation between implicit self-esteem and subjective well-being (Schimmack and Diener, 2003). In individuals with low explicit self-esteem, implicit self-esteem did not affect individuals’ subjective well-being (Xu et al., 2005). The divergence in these few studies may be the moderating effect of self-threat. Both self-esteem and well-being are overall evaluative variables. Well-being involves general judgments about one’s life, and self-esteem involves broad judgments about oneself. Thus, a complex relationship between self-esteem and well-being was presented (Xu et al., 2005).

The relationship between affect and self-esteem is influenced by implicit theory. Implicit theory suggests that people perceive personal qualities such as intelligence, motor ability, social skills, and personality traits differently (Dweck et al., 1995). Researchers have proposed systematic differences in people’s implicit affect regulation theories: some people prefer to view affect as fixed (Entity Theory). In contrast, others view affect as more malleable (Incremental Theory), use more adaptive self-regulatory behaviors in the face of challenges to increase their success, and have higher levels of self-esteem (Tamir et al., 2013). This may also explain why many studies have shown that positive affect is significantly and positively related to self-esteem (Tarlow and Haaga, 1996; Zhang and Zheng, 2004); conversely, negative affect is significantly negatively related to self-esteem (Dua, 1993; Tarlow and Haaga, 1996). In other words, individuals who are good at regulating their emotional state and self-esteem are more likely to experience positive affect. Specifically, individuals who are good at handling their emotional state value positive affect (Wood et al., 2003) and tend to experience less negative affect (Smith and Petty, 1995). Individuals who are good at regulating emotional states value positive affect, tend to reduce negative affect, and tend to have higher levels of self-esteem. Dynamic automation refers to the fact that emotional processing is rapid and unregulated by attention and consciousness (Wang et al., 2016). Therefore, in the mediation between current performance and self-esteem, the automatic processing of affect may take precedence over the processing of self-esteem. Hence, we proposed H5.

*H5*: Affect and self-esteem play a serial mediation role between current performance in the task and predicted well-being.

### 1.4. The present study

This study was conducted to experimentally investigated the impact of current failures on the evaluation of future goals. We aimed to explore different mechanisms of action in high and low self-threat situations. We report two studies focusing on these ideas. Using a between-group design, participants in each experiment were randomly assigned performance feedback. Study 1 as a pilot study tested the effect of current failures on the predicted well-being by giving participants randomized performance feedback on an intelligence test (H1). Study 2 examined the validity of the two hypotheses [“grasping the nettle (H2)” or “belittling the goal (H3)”] in a high and low self-threat situation. In addition, the purpose of Study 2 is to explore the moderating role of situational self-threat in the relationship between current performance and predicted well-being (H4) and the serial mediation role of current affect and self-esteem (H5). We confirm that we report all data exclusions, all measures and all manipulations in the two studies.

## 2. Study 1

### 2.1. Method

#### 2.1.1. Participants

The experiment was conducted with 197 participants recruited online. Data from participants appearing in either of the following two situations were excluded: (1) participants with prior training related to weight and quantity estimation; (2) participants who gave highly consistent ratings on all items. The retained sample comprised 168 participants, with a valid recovery rate of 85.28%. Among them, 89 were male, and 88 were female, with a mean age of 19.87 years (SD = 4.17; range 18–50).

The study used Gpower3.1 for *post hoc* statistical test efficacy analysis (Cunningham and McCrum-Gardner, 2007; Faul et al., 2009). With the middle meaningful effect size (*w* = 0.30) and *α* = 0.05, the results showed the detectable statistical test efficacy for the current sample (*N* = 168) of this study was 0.973, indicating that the sample size of this study was adequate.

#### 2.1.2. Research tools

Experimental materials include:

(1) The weight estimation test (Test A) had six questions, such as, “Please estimate the whale’s weight in the picture.” (2) The quantity estimation test (Test B) had six questions, such as, “Please estimate the number of leaves in the picture.” Participants were informed that all answers within ±20% were considered correct. Since estimating the exact answer was almost impossible, making the manipulation of controlling for in-the-moment performance feedback is relatively unquestionable. (3) For the measure of predicted well-being, participants were required to rate their level of predicted well-being (out of 100) for achieving a high score on either Test A or Test B after retaking the tests, such as, “Please predict how predicted well-being you will be of with a high score (in the top 10% of the population) when you take a Test A again? (out of 100)”

#### 2.1.3. Research procedures

Permission to conduct the study was granted by the Research Ethics Committee at the institution with which the first author was affiliated. First, the purpose and requirements of the study were explained, and then participants indicated their informed consent. Participants were randomly assigned to two feedback conditions (Good scores on test A and poor scores on test B; Poor scores on test A and good scores on test B).

Before the experiment began, the text “This is a fun quiz” appeared, and participants were told that all answers within ±20% were considered correct. After the participants completed Test A and Test B, they were given different scores according to their experiment conditions and asked to remember their scores. Subsequently, they rated their well-being (out of 100) in terms of their having achieved a high score on Test A or Test B after retaking the examination.

We conducted a manipulation check in which participants assessed the difficulty of achieving a high score on each of Test A and Test B (0 = “not at all,” 10 = “very much”), which aimed to ensure that their perceived level of difficulty was related to the test feedback they received. Finally, we thanked the participants.

### 2.2. Results and analysis

#### 2.2.1. Manipulation validity test

The participants scored higher on the perceived difficulty for poorer grades and lower on the perceived difficulty for better grades (*M*_poor score_ = 8.74, SD_poor score_ = 2.01 vs. *M*_high score_ = 6.28, SD_high score_ = 2.68, *t*(167) = 15.96, *p* = 0.000, *d* = 1.04) on the same questionnaire. Here *d* > 0.8 indicates that there is a significant difference in feedback between the two experimental conditions, and the effect is relatively high. There was a significant difference in the perceived difficulty of the questionnaire. Therefore, the context creation was valid, and the degree of difficulty perceived by the participants was related to the feedback received.

#### 2.2.2. Testing the effect of current performance on predicted well-being

This study conducted a chi-square test with 2 different feedback conditions (better performance on Test A and worse performance on Test B, worse performance on Test A and better performance on Test B) × 2 (prediction level of well-being: higher, lower) column table. The chi-square test results indicate that $\chi^{2}$ (1,*N* = 168) = 22.83, *p*<0.001,meaning that the predicted well-being levels differed across the feedback conditions. With bad feedback, the individual’s predicted well-being of future performance was high. In the other situations, the individual’s predicted well-being of future performance in the tests was low.

### 2.3. Discussion

In Study 1, there was a significant difference in the predicted well-being of individuals to perform well in the task in the future between these two experimental conditions. With good feedback, the individual’s predicted well-being of future performance in the tests was low. With bad feedback, the individual’s predicted well-being of future performance was high. It means that individuals have a higher predicted well-being of future success after an unimportant loss, confirming H1. However, the better feedback may maintain the individual’s self-esteem and interfere with the effect of poorer feedback on self-esteem, meaning maybe H2 is confirmed. Thus, in Study 2, participants were randomly assigned to high and low self-threat situations with a larger group of participants and relatively independent experiments.

## 3. Study 2

### 3.1. Methods

#### 3.1.1. Participants

The experiment was conducted with 524 participants recruited online. Data from participants who appeared in either of the following two situations were removed: (1) participants with prior training related to weight and quantity estimation; (2) participants who gave highly consistent ratings on all items. A total of 407 valid data were recovered, with a valid recovery rate of 77.67%. Among them, 225 and 182 were male and female, respectively, with a mean age of 24.75 years (SD = 5.92; range 18–66).

#### 3.1.2. Research tools

The threat initiation material included 12 intellectual threat test questions on quantity and weight estimation. Participants were told that all answers within ±20% of the range are considered correct, making the false feedback manipulation relatively unquestionable since estimating the exact answer is nearly impossible.

The Rosenberg Self-Esteem Scale (RSES) is a scale designed to assess individuals’ overall feelings about self-worth and self-acceptance (Rosenberg, 1965; Wang et al., 1999). The scale consists of 10 items rated on four levels, with positive and negative scores and a total score range of 10–40, with higher scores indicating higher self-esteem. This scale has been widely used. It is concise, easy to score, and directly assesses participants’ positive or negative feelings. Examples of these feelings include: “I feel that I have many good qualities,” and “I wish I could earn more respect for myself.” In this study, Cronbach’s alpha coefficient for the entire scale was 0.727, indicating good reliability for subsequent data analysis. Therefore, we could use the scales in further analyses.

The Index of General Affect of the Campbell Index of Well-being was used (Cohn et al.,1977; Wang et al., 1999; Ryff et al., 2014). The Campbell Index of Well-being consists of the Index of General Affect and the life satisfaction subscale. The Index of General Affect is used to measure the affect currently experienced by the participant. It consists of eight items that describe the connotation of affect from different perspectives on a seven-point scale, with positive and negative scoring. For example, 1 indicates happiest and 7 most miserable. The total score ranges from 7 to 56, with a higher total indicating a more positive affect. In this study, Cronbach’s alpha coefficient for the entire scale was 0.730, indicating good reliability for subsequent data analysis. Therefore, we could use the scales in further analyses.

The Predicted Well-Being Measure asked the question, “Please predict how well-being you will be of with a high score (in the top 10% of the population) when you take a new test again? (out of 10)” This was used to measure predicted well-being.

#### 3.1.3. Research procedures

Permission to conduct the study was granted by the Research Ethics Committee at the institution with which the first author was affiliated. First, the purpose and requirements of the study were explained, and then participants indicated their informed consent. Participants were randomly assigned to experiment conditions in two contexts, one with the text “There are two fun tests in this task that you need to estimate based on your perceptions.” The other text is “This test can effectively measure individual intelligence and ability level. The more questions you get right, the higher your intelligence and ability, and the more likely you will achieve a high degree of success and well-being. You will need to use your skills to estimate and answer as soon as possible.”

After completing the 12 test questions on quantity estimation and weight estimation, the participant was given false score feedback with the following three results: “Congratulations, you answered 10 out of 12 questions correctly and scored in the top 15% of the population, which indicates a perfect score,” “You answered 6 out of 12 questions correctly and outscored around 50% of the people, which means an average score,” or “Unfortunately, you answered 2 out of 12 questions correctly and scored in the bottom 15% of the population, which indicates a very low score.”

Participants completed the RSES, the Affect subscale, and a single question on predicted well-being.

We conducted a manipulation check to allow participants to assess the difficulty of achieving a high score on the task (0 = “not at all,” 10 = “very much”) to ensure that their perceived level of difficulty was related to the test feedback they received. Finally, we thanked the participants.

### 3.2. Results and analysis

#### 3.2.1. Common method deviation control

The KMO value was 0.904 and Bartlett’s spherical test value was significantly 0.000 (Sig. < 0.001), making the data suitable for exploratory factor analysis. Since the prerequisites for factor analysis were met, the Harman one-way test was used for statistical testing of factor analysis. There were five principal components with eigenvalues greater than 1; their cumulative contribution rate reached 64.15%; the variance explained by the first principal component was 27.62%; and there was no factor with excessive explanatory power. Thus, the common method bias of this study was within a reasonable range, and the questionnaire had good structural validity.

#### 3.2.2. Manipulation validity test

Participants who performed better were more likely to perceive the test as less difficult, followed by participants who performed moderately [*M*_high score_ = 6.32, SD_high score_ = 2.72 vs. *M*_medium score_ = 7.49, SD_medium score_ = 2.62, *t* (266) = −3.57, *p* < 0.001, *d* = 0.44]. Here 0.2 < *d* < 0.5 indicates that there is a significant difference in feedback between the two experimental conditions, and the effect is medium. The participants who performed worse were more likely to perceive the test as more difficult [*M*_high score_ = 6.32, SD_high score_ = 2.72 vs. *M*_poor score_ = 8.17, SD_poor score_ = 2.35, *t* (276) = −6.066, *p* < 0.001, *d* = 0.73]. Here 0.5 < *d* < 0.8 indicates that there is a significant difference in feedback between the two experimental conditions, and the effect is relatively high. Therefore, participants had lower perceived difficulty scores for better performance and higher perceived difficulty scores for worse performance. Subsequent multiple comparisons of the different feedback experiment conditions and ANOVA results showed that there was a significant difference in feedback between the experiment conditions (*F* = 18.425, *p* < 0.001) and a significant difference in feedback between two different experiment conditions (*p* < 0.05). Thus, context creation was effective, and the level of difficulty perceived by the participants was related to the feedback received.

#### 3.2.3. Descriptive statistics and correlation comparisons

The mean, standard deviation, and Pearson correlation matrix of each variable in low self-threat situation are shown in Table 1. The correlation analysis revealed that current performance was not significantly correlated with affect, self-esteem, and predicted well-being; a significant positive correlation was found between affect, self-esteem, and predicted well-being. Gender and age were not significantly correlated with affect, self-esteem, and predicted well-being. The correlations among the variables could not support the subsequent model. Although it is not significant, current performance and predicted well-being are positively correlated, indicating that more people have higher predicted well-being after failure in low self-threat situation, which may support H2 (the grasp-the-nettle hypothesis). It needs to be explored further.

**Table 1:** Means, standard deviations and correlations of variables in low self-threat situation (*N* = 198).

Variables	*M*	SD	1	2	3	4	5
1 Gender	−	−	−				
2 Age	25.07	7.194	−0.107	−			
3 Current performance	49.82	29.187	−0.239^***^	0.163^*^	−		
4 Affect	4.60	1.137	0.071	0.055	−0.057	−	
5 Self-esteem	28.83	5.005	0.067	0.100	−0.010	0.790^***^	−
6 Predicted well-being	8.74	1.935	−0.039	0.098	0.043	0.213^**^	0.308^***^

*N* = 198, *p < 0.05, **p < 0.01, ***p < 0.001.

The mean, standard deviation, and Pearson correlation matrix of each variable in high self-threat situation are shown in Table 2. The correlation analysis revealed that current performance was significantly negatively correlated with affect, self-esteem, and predicted well-being; a significant positive correlation was found between affect, self-esteem, and predicted well-being. Age was significantly correlated with affect, self-esteem, and predicted well-being, and age would be used as a control variable in the subsequent analysis. Gender was not significantly correlated with affect, self-esteem, and predicted well-being. The correlations among the variables support the subsequent hypothesis testing. Controlling for age, current performance significantly and negatively predicted well-being, *β* = −0.227, *p* = 0.001, *R* = 0.28, for the model *ΔR*^2^ = 0.08, *F* (4,204) = 7.95, *p* = 0.000. The result supports H3 (the belittle-the-goal hypothesis).

**Table 2:** Means, standard deviations and correlations of variables in high self-threat situation (*N* = 209).

Variables	*M*	SD	1	2	3	4	5
1 Gender	−	−	−				
2 Age	24.45	4.366	−0.043	−			
3 Current performance	50.17	28.816	−0.040	0.005	−		
4 Affect	4.97	1.071	0.001	0.216^**^	−0.366^***^	−	
5 Self-esteem	30.13	4.845	−0.018	0.174^*^	−0.256^**^	0.652^***^	−
6 Predicted well-being	8.87	1.802	−0.133	0.142^*^	−0.246^*^	0.328^***^	0.324^***^

*N* = 209, *p < 0.05, **p < 0.01, ***p < 0.001.

#### 3.2.4. Test of the effect of situational self-threat on predicted well-being

The top-ranking participants were considered as the success experimental condition, and the low-ranking participants were considered as the failure experimental condition. The top-ranking participants in the high self-threat situation had higher predicted well-being, and the top-ranking participants in the low self-threat situation had lower predicted well-being [*M*_high self-threat_ = 9.50, SD_high self-threat_ = 1.38 vs. *M*_low self-threat_ = 8.45, SD_low self-threat_ = 1.95, *t* (137) =36.09, *p* < 0.001, *d* = 0.62], indicating that there is a significant difference in feedback between the two experimental conditions, and the effect is relatively high. There was no significant difference in the predicted well-being of the low-ranking participants in the high and low self-threat situations [*M*_high self-threat_ = 8.42, SD_high self-threat_ = 2.09 vs. *M*_low self-threat score_ = 8.65, SD_low self-threat_ = 1.96, *t* (137) =35.68, *p* < 0.001, *d* = 0.11]. Thus, individuals have a higher predicted well-being of future success in a low self-threat situation, which supports H2; individuals have a lower predicted well-being of future success in a high self-threat situation, which supports H3.

#### 3.2.5. Mediating effect test

**Figure 1:**
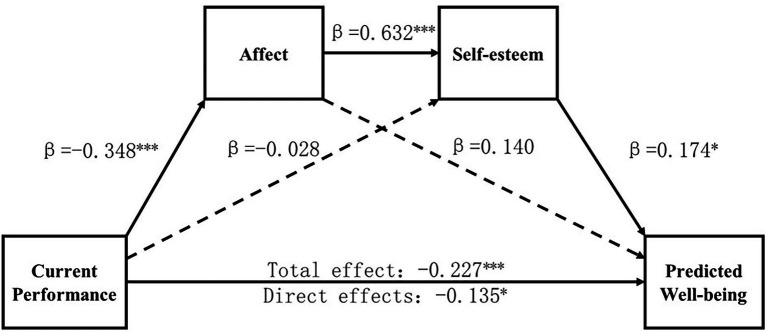
Chain intermediary model in high self-threat situation (*N* = 209). *p<0.05, ***p<0.001.

SPSS plug-in PROCESS model 6 was used for high and low self-threat situations. Current performance was presented as the independent variable and predicted well-being as the dependent variable. Affect and self-esteem were presented as serial mediator variables, and age as the control variable. The serial mediation model results in high self-threat situations shown in Figure 1.

The regression equation was significant in the high self-threat situation, *R*^2^ = 0.15, *F* (4,204) =9.24, *p < 0*.001.95% confidence intervals for mediating effects were estimated by the Bootstrap sampling method with 5,000 sample draws. As shown in Table 3, the results indicated that the total effect was −0.227 (*p* < 0.001), the direct effect was −0.135 (*p* < 0.05), and the confidence intervals for the mediating variables contained 0 values. This indicates that the indirect effect from the path was not significant. The indirect effect of the path mediated by affect and self-esteem was −0.038 (95% CI = [−0.084, −0.004]), indicating that the serial mediation effect of affect and self-esteem in the negative effect of current performance predicted well-being holds true. The result supports H5. In low self-threat situation, the regression equation is not significant. The significant difference between the models in the high and low self-threat contexts suggests that the self-threat situations moderate the relationship between current performance and predicted well-being, a result that tentatively supports H4.

**Table 3:** Analysis of intermediary effects.

	Indirect effect value	Bootstrap SE	Boot CI lower limit	Boot CI upper limit	Relative mediating effect
Total indirect effect	−0.092	0.035	−0.167	−0.030	40.53%
Indirect effect 1	−0.049	0.039	−0.131	0.025	
Indirect effect 2	−0.005	0.012	−0.035	0.013	
Indirect effect 3	−0.038	0.020	−0.084	−0.004	16.74%

Boot standard error, the lower limit of Boot CI, and the upper limit of Boot CI refer to the lower and upper limits of the standard error of the profile effect, 95% confidence interval estimated by the percentile Bootstrap method of bias correction, respectively.

#### 3.2.6. Moderated mediation analysis

The structural equation model confirmatory factor analysis (CFA) was conducted for the total model, the high and low self-threat situation model. CFA has the advantage of producing goodness of fit indices that allows for comparison of multi-cluster comparison analysis and autoregressive path model. As shown in Table 4, the results show that the three models fit well, and the high self-threat situation model fit is the best ( $\chi^{2}/\mathrm{df}$ = 1.771, RMSEA = 0.061).

**Table 4:** Structural equation model confirmatory factor analysis.

Models	$\chi^{2}$	df	$\chi^{2}/df$	CFI	TLI	RMSEA	SRMR
Total Model	47.093	19	2.479	0.985	0.977	0.060	0.028
High Self-threat Situation Model	33.656	19	1.771	0.984	0.976	0.061	0.004
Low Self-threat Situation Model	37.475	19	1.972	0.980	0.970	0.070	0.033

$\chi^{2}$ */*df < 3, CFI > 0.90, TLI > 0.90, RMSEA < 0.07, SRMR < 0.08 indicates that the model fits well.

A multi-cluster comparison of structural equation models was conducted to determine whether the proposed model applies to high and low self-threat situations. The critical values of differences between standardized path coefficients and parameters among current performance, affect, self-esteem, and predicted well-being in high and low self-threat situations are shown in Table 5. When the critical value of the difference is less than 1.96 (significant level of 0.05), it indicates no significant difference. Five paths (affect → self-esteem, current performance → self-esteem, current performance → predicted well-being, affect → predicted well-being, and self-esteem → predicted well-being) do not differ significantly between the high and low self-threat situations. In contrast, the current performance → affect path varies considerably between the high and low self-threat situations, indicating that the self-threat situations moderate this path. Situational self-threat plays a moderating role in the model, which supported H4 again.

**Table 5:** Comparison of differences in path coefficients of high and low self-threat clusters.

Paths	The high self-threat situation	The low self-threat situation	Variance Threshold	Differential results
Current moment performance → Affect	−0.352	−0.058	−3.105	Yes
Affect → Self-esteem	0.657	0.786	−1.779	No
Current performance → Self-esteem	−0.019	0.035	−0.775	No
Current performance → Predicted well-being	−0.145	0.037	−1.890	No
Affect → Predicted well-being	0.136	−0.073	1.460	No
Self-esteem → Predicted well-being	0.177	0.369	−1.367	No

All coefficients are unstandardized; When the critical value of the difference is less than 1.96 (*p* > 0.05), it indicates no significant difference; When the critical value of the difference is more than 1.96 (*p* < 0.05), it indicates there is a significant difference.

## 4. General discussion

### 4.1. Conclusion

In Study 1, with good feedback, the individual’s predicted well-being of future performance in the tests was low; with bad feedback, the individual’s predicted well-being of future performance was high. It means that individuals have a higher predicted well-being of future success after an unimportant loss, confirming H1.

Study 2 shows individuals have a higher predicted well-being of future success in a low self-threat situation (supporting H2) and a lower predicted well-being of future success in a high self-threat situation (supporting H3). Situational self-threat plays a moderating role between current performance and predicted well-being, confirming H4. The serial mediation role of affect and self-esteem in the negative effect of current performance on predicted well-being holds in high self-threat situations, supporting H5.

### 4.2. Research contributions

#### 4.2.1. Current performance and self-threat situations jointly influence predicted well-being

The current research testifies to the influence of Current performance and self-threat situations on the predicted well-being, which expands the research on self-threat. In present study, the high self-threat situation was set up as a highly relevant and competitive ability test for one’s future; the low self-threat situation was set up as an irrelevant game of feelings. The present study has the same results as previous studies: a stimulus can be perceived as a source of threat depends on the specific context (Liu et al., 2017); when people analyze objectively without experiencing the pain of failure themselves, they do not anticipate the defensive reactions of the actual participants (Sjstad et al., 2020; Luan and Li, 2022). In other words, current performances and the self-threat situations work together to build a complex self-threat model, producing an integrative model of the related mental mechanism and a new theoretical perspective. The individual constructs the self-threat level judgment based on the current assessment and adjusts the future goal by combining the threat level of the situation and the threat level of the current performance.

#### 4.2.2. The path of chain intermediaries

The insignificant path of the single mediating variable of affect and self-esteem and the significant serial mediation path deserve discussion. In present study, The Index of General Affect Scale can reflect the short-term affect fluctuation of the participants after receiving the current performance (Cohn et al.,1977; Wang et al., 1999; Ryff et al., 2014); The Rosenberg Self-Esteem Scale is usually used to measure explicit self-esteem (Rosenberg, 1965; Wang et al., 1999). Research on the predictive effects of implicit and explicit self-esteem on subjective well-being is divergent (Schimmack and Diener, 2003; Xu et al., 2005). Implicit mental activity influences individuals’ behavioral responses (Greenwald and Farnham, 2000). These differences in the results of inferential self-esteem studies also reflect the complexity of implicit processing, which has the same results as previous studies. The implicit processing of affect takes precedence over external self-esteem processing. Therefore, the paths of the single mediating variable of affect and self-esteem were insignificant and the serial mediation path was significant. This research expands the literature on development of appropriate cognitive theories by specifying the behavioral consequences and analyzing the psychological process in high and low self-threat situations.

#### 4.2.3. Practical implications of enhancing predicted well-being

When experiencing failure in real life, this mental mechanism in high and low self-threat situations is universally applicable. Therefore, this mechanism can also provide suggestions on how to improve the predicted well-being to better deal with failure. How to reduce the negative impact of failure on individuals in high self-threat situation and make individuals rise to the challenge? In high self-threat situations, shifting efforts to different, more attainable goals may improve well-being (Davidai and Deri, 2019); this prevents people from wasting time and energy on useless pursuits or disengaging from unattainable goals (Wrosch et al., 2016). Present study inspires us getting success in other events or disengaging from this goal through timely stop-loss are good ways to keep a higher level of affect and self-esteem after experiencing initial failure. In low self-threat situations, Overestimating the prospective affect can encourage people to work harder toward an achievable goal (Baumeister et al., 2007; Miloyan and Suddendorf, 2015). In present study, having a sensible plan as one’s future goal is more likely to produce higher predicted well-being and thus motivates effort. These novel measures of dealing with failure in high and low self-threat situations may boost predicted well-being and actual well-being.

### 4.3. Future research

In addition to the above contributions, we acknowledge that the present study has certain limitations that can be further explored in future studies. First, the study predominantly explored predicted well-being in high and low self-threat situations but did not address the future experience of actual well-being. Therefore, future studies must increase the detection of actual well-being to improve the mechanism of the effect of self-threat on actual well-being with a more extended period. In addition, one limitation of this research is the possible selection bias: It analyzed only those participants under self-threat because of performance and situation. However, the results cannot reflect the participants who got success. It is different that people react after experiencing a game success or a real success. Future research can take a further step to explore the reactions of participants after a current success.

## Data availability statement

The original contributions presented in the study are included in the article/supplementary material, further inquiries can be directed to the corresponding author.

## Ethics statement

The studies involving human participants were reviewed and approved by Institution of Psychology, China University of Geosciences, Wuhan. The patients/participants provided their written informed consent to participate in this study.

## Author contributions

ZH proposed research questions, wrote and revised the manuscript. YW designed the study, collected and analyzed the data, wrote and revised the manuscript. LL designed the study, analyzed the data, and wrote the manuscript. JS designed the study, analyzed the logic of the hypotheses, and wrote the manuscript. All authors contributed to the article and approved the submitted version.

## Funding

The research was supported by the MOE (Ministry of Education in China) Project of Humanities and Social Sciences (Project No. 18YJA710014).

## Conflict of interest

The authors declare that the research was conducted in the absence of any commercial or financial relationships that could be construed as a potential conflict of interest.

## Publisher’s note

All claims expressed in this article are solely those of the authors and do not necessarily represent those of their affiliated organizations, or those of the publisher, the editors and the reviewers. Any product that may be evaluated in this article, or claim that may be made by its manufacturer, is not guaranteed or endorsed by the publisher.

## References

[ref1] Arnold M. B. (1960). Emotion and personality. Am. J. Psychol. 76:516. doi: 10.2307/1419805

[ref2] Baumeister R. Chester F. David S. Brad B. Dewall J. (2016). How often does currently felt emotion predict social behavior and judgment? A meta-analytic test of two theories. Emot. Rev. 8, 136–143. doi: 10.1177/1754073915572690

[ref3] Baumeister R. Vohs F. Kathleen D. Dewall C. Zhang N. (2007). How emotion shapes behavior: feedback, anticipation, and reflection, rather than direct causation. Pers. Soc. Psychol. Rev. 11, 167–203. doi: 10.1177/1088868307301033 18453461

[ref4] Baumeister R. F. Maranges H. M. Sjåstad H. (2018). Consciousness of the future as a matrix of maybe: pragmatic prospection and the simulation of alternative possibilities. Psychol. Conscious. Theory Res. Pract. 5, 223–238. doi: 10.1037/cns0000154

[ref5] Blascovich J. Seery M. D. Mugridge C. A. Norris R. K. Weisbuch M. (2004). Predicting athletic performance from cardiovascular indexes of challenge and threat. J. Exp. Soc. Psychol. 40, 683–688. doi: 10.1016/j.jesp.2003.10.007

[ref6] Blascovich J. Tomaka J. (1996). The biopsychosocial model of arousal regulation. Adv. Exp. Soc. Psychol. 28, 1–51. doi: 10.1016/S0065-2601(08)60235-X

[ref7] Butler A. C. Hokanson J. E. Flynn H. A. (1994). A comparison of self-esteem lability and low trait self-esteem as vulnerability factors for depression. J. Pers. Soc. Psychol. 66, 166–177. doi: 10.1037/0022-3514.66.1.166, PMID: 8126646

[ref8] Chen L. Qu L. (2021). From stressful experiences to depression in Chinese migrant children: the roles of stress mindset and coping. Front. Psychol. 12:868. doi: 10.3389/fpsyg.2021.601732, PMID: 33889105 PMC8056082

[ref55] Cohn Richard M. Campbell Angus, Converse Philip E. Rodgers Willard L. (1997). The Quality of American Life: Perceptions, Evaluations, and Satisfactions. Contemporary Sociology, 4. doi: 10.2307/2066476

[ref9] Cunningham J. B. McCrum-Gardner E. (2007). Power, effect and sample size using GPower: practical issues for researchers and members of research ethics committees. Evid. Based Midwifery 5, 132–136.

[ref10] Davidai S. Deri S. (2019). The second pugilist’s plight: why people believe they are above average but are not especially happy about it. J. Exp. Psychol. Gen. 148, 570–587. doi: 10.1037/xge0000580, PMID: 30802129

[ref11] Diener E. (2000). Subjective well-being. The science of happiness and a proposal for a national index. Am. Psychol. 55, 34–43. doi: 10.1037/0003-066X.55.1.34 11392863

[ref12] Dimenichi B. C. Richmond L. L. (2015). Reflecting on past failures leads to increased perseverance and sustained attention. J. Cogn. Psychol. 27, 180–193. doi: 10.1080/20445911.2014.995104

[ref13] Dua J. K. (1993). The role of negative affect and positive affect in stress, depression, self-esteem, assertiveness, type a behaviors, psychological health, and physical health. Genet. Soc Gen. Psychol. Monogr. 119, 515–552. PMID: 8150272

[ref14] Dutton K. A. Brown J. D. (1997). Global self-esteem and specific self-views as determinants of People’s reactions to success and failure. J. Pers. Soc. Psychol. 73, 139–148. doi: 10.1037/0022-3514.73.1.139

[ref15] Dweck C. S. Chiu C. Y. Hong Y. Y. (1995). Implicit theories and their role in judgments and reactions: a word from two perspectives. Psychol. Inq. 6, 267–285. doi: 10.1207/s15327965pli0604_1

[ref16] Eskreis-Winkler L. Fishbach A. (2019). Not learning from failure-the greatest failure of all. Psychol. Sci. 30, 1733–1744. doi: 10.1177/0956797619881133, PMID: 31702452

[ref17] Eskreis-Winkler L. Fishbach A. (2022). You think failure is hard? So is learning from it. Perspect. Psychol. Sci. 17, 1511–1524. doi: 10.1177/17456916211059817, PMID: 35580276

[ref18] Faul F. Erdfelder E. Buchner A. Lang A. G. (2009). Statistical power analyses using g∗power 3.1: tests for correlation and regression analyses. Behav. Res. Methods 41, 1149–1160. doi: 10.3758/brm.41.4.1149, PMID: 19897823

[ref19] Feather N. T. (1966). Effects of prior success and failure on expectations of success and subsequent performance. J. Pers. Soc. Psychol. 3, 287–298. doi: 10.1037/h0022965, PMID: 5906331

[ref20] Festinger L. (1954). A theory of social comparison processes. Hum. Relat. 7, 117–140. doi: 10.1177/001872675400700202

[ref21] Greenberg J. Solomon S. Pyszczynski T. Rosenblatt A. Burling J. Lyon D. (1992). Why do people need self-esteem? Converging evidence that self-esteem serves an anxiety-buffering function. J. Pers. Soc. Psychol. 63, 913–922. doi: 10.1037/0022-3514.63.6.913, PMID: 1460559

[ref22] Greenwald A. G. Farnham S. D. (2000). Using the implicit association test to measure self-esteem and self-concept. J. Pers. Soc. Psychol. 79, 1022–1038. doi: 10.1037/0022-3514.79.6.I022 11138752

[ref23] Hakmiller K. L. (1966). Threat as a determinant of downward comparison. J. Exp. Soc. Psychol. 1, 32–39. doi: 10.1016/0022-1031(66)90063-1

[ref24] Han X. Chi Y. (2012). The self-threat of unsolicited social comparison and its balance. Acta Psychol. Sin. 44, 1628–1640. doi: 10.3724/SP.J.1041.2012.01628

[ref25] Jamieson J. P. Hangen E. J. Lee H. Y. Yeager D. S. (2018). Capitalizing on appraisal processes to improve affective responses to social stress. Emot. Rev. 10, 30–39. doi: 10.1177/1754073917693085, PMID: 31178923 PMC6550483

[ref26] Liu M. Wu S. Wang Y. Zhang X. Yang J. (2017). Self-threat and defense: the modulation effect of self-esteem. Psychol. Techniques Appl. 5, 43–51. doi: 10.16842/j.cnki.issn2095-5588.2017.01.006

[ref27] Luan M. Li J. (2022). Failed players, successful advertisements: does showing the failure experience increase observers’ intention to try? Acta Psychol. Sin. 54, 1562–1578. doi: 10.3724/SP.J.1041.2022.01562

[ref28] Mcmahan I. D. (1973). Relationships between causal attributions and expectancy of success. J. Pers. Soc. Psychol. 28, 108–114. doi: 10.1037/h0035474

[ref29] Miloyan B. Suddendorf T. (2015). Feelings of the future. Trends Cogn. Sci. 19, 196–200. doi: 10.1016/j.tics.2015.01.008 25726365

[ref30] Morewedge C. K. Buechel E. C. (2013). Motivated underpinnings of the impact bias in affective forecasts. Emotion 13, 1023–1029. doi: 10.1037/a0033797, PMID: 23914762

[ref31] Newman D. B. Nezlek J. B. (2022). The influence of daily events on emotion regulation and well-being in daily life. Personal. Soc. Psychol. Bull. 48, 19–33. doi: 10.1177/0146167220980882, PMID: 33504280

[ref32] Oettingen G. Marquardt M. K. Gollwitzer P. M. (2012). Mental contrasting turns positive feedback on creative potential into successful performance. J. Exp. Soc. Psychol. 48, 990–996. doi: 10.1016/j.jesp.2012.03.008

[ref33] Paradise A. W. Kernis M. H. (2002). Self-esteem and psychological well-being: implications of fragile self-esteem. J. Soc. Clin. Psychol. 21, 345–361. doi: 10.1521/jscp.21.4.345.22598

[ref34] Qu L. Lim Z. M. (2016). Adults’ descriptions of a situation can influence children’s appraisal, feelings, and subsequent psychological functions. Child Dev. 87, 1550–1563. doi: 10.1111/cdev.12540, PMID: 27089833

[ref35] Rosenberg M. (1965). Rosenberg self-esteem scale (RSE): acceptance and commitment therapy. Measur. Pack 61:52.

[ref36] Rosenberg M. Schooler C. Schoenbach C. Rosenberg F. (1995). Global self-esteem and specific self-esteem: different concepts, different outcomes. Am. Sociol. Rev. 60, 141–156. doi: 10.2307/2096350

[ref37] Ryff C. D. (2014). Culture and the Promotion of Well-being in East and West: Understanding Varieties of Attunement to the Surrounding Context. In: Fava, G., Ruini, C. (eds) Increasing Psychological Well-being in Clinical and Educational Settings. Cross-Cultural Advancements in Positive Psychology, *vol 8*. Springer, Dordrecht. doi: 10.1007/978-94-017-8669-0_1

[ref56] Shepperd J. A. Findley-Klein C. Kwavnick K. D. Walker D. Perez S. (2000). Bracing for loss. J. Pers. Soc. Psychol. 78, 620–634., PMID: 10794370 10.1037//0022-3514.78.4.620

[ref38] Schimmack U. Diener E. (2003). Predictive validity of explicit and implicit self-esteem for subjective well-being. J. Res. Pers. 37, 100–106. doi: 10.1016/S0092-6566(02)00532-9

[ref39] Sedikides C. (1993). Assessment, enhancement, and verification determinants of the self-evaluation process. J. Pers. Soc. Psychol. 65:317. doi: 10.1037/0022-3514.65.2.317

[ref40] Sjstad H. Baumeister R. F. Ent M. (2020). Greener grass or sour grapes? How people value future goals after initial failure. J. Exp. Soc. Psychol. 88:103965. doi: 10.1016/j.jesp.2020.10396

[ref41] Smith S. M. Petty R. E. (1995). Personality moderators of mood congruency effects on cognition: the role of self-esteem and negative mood regulation. J. Pers. Soc. Psychol. 68, 1092–1107. doi: 10.1037/0022-3514.68.6.1092, PMID: 7608856

[ref42] Steele C. M. (1988). The psychology of self-affirmation: sustaining the integrity of the self. Adv. Exp. Soc. Psychol. 21, 261–302. doi: 10.1016/S0065-2601(08)60229-4

[ref43] Suhail K. Chaudhry H. R. (2004). Predictors of subjective well-being in an eastern Muslim culture. J. Soc. Clin. Psychol. 23, 359–376. doi: 10.1521/jscp.23.3.359.35451

[ref44] Tamir M. John O. P. Srivastava S. Gross J. J. (2013). Implicit theories of emotion: affective and social outcomes across a major life transition. J. Pers. Soc. Psychol. 92, 731–744. doi: 10.1037/0022-3514.92.4.731 17469955

[ref45] Tarlow E. M. Haaga D. (1996). Negative self-concept: specificity to depressive symptoms and relation to positive and negative affectivity. J. Res. Pers. 30, 120–127. doi: 10.1006/jrpe.1996.0008

[ref46] Vandellen M. R. Campbell W. K. Hoyle R. H. Bradfield E. K. (2011). Compensating, resisting, and breaking: a meta-analytic examination of reactions to self-esteem threat. Pers. Soc. Psychol. Rev. 15, 51–74. doi: 10.1177/1088868310372950, PMID: 20631397

[ref47] Wang L. Jia L. Luo Y. (2016). Automatic processing of emotions: evidence and controversy. Adv. Psychol. Sci. 24, 1185–1197. doi: 10.3724/SP.J.1042.2016.01185

[ref48] Wang X. D. Wang X. L. Ma H. (1999). Rating Scales for Mental Health. Beijing: Mental Health Magazine Press.

[ref49] Wilson T. D. Wheatley T. P. Kurtz J. L. Dunn E. W. Gilbert D. T. (2004). When to fire: anticipatory versus postevent reconstrual of uncontrollable events. Personal. Soc. Psychol. Bull. 30, 340–351. doi: 10.1177/0146167203256974, PMID: 15510418

[ref50] Wood J. V. Heimpel S. A. Michela J. L. (2003). Savoring versus dampening: self-esteem differences in regulating positive affect. J. Pers. Soc. Psychol. 85, 566–580. doi: 10.1037/0022-3514.85.3.566, PMID: 14498791

[ref51] Wrosch C. Scheier M. F. Miller G. E. Schulz R. Carver C. S. (2016). Adaptive self-regulation of unattainable goals: goal disengagement, goal reengagement, and subjective well-being. Personal. Soc. Psychol. Bull. 29, 1494–1508. doi: 10.1177/0146167203256921, PMID: 15018681

[ref52] Xu W. Wu M. Qiu F. (2005). A research on the relationship between self-esteem and subjective well-being. Psychol. Sci. 03, 562–565. doi: 10.16719/j.cnki.1671-6981.2005.03.012

[ref53] Zhang L. Wang B. Feng X. Zhang Y. Wang W. (2022). Exploring the influence of failure aversion on learning from project failure: a Sensemaking perspective. Front. Psychol. 13:794390. doi: 10.3389/fpsyg.2022.794390, PMID: 35592145 PMC9110797

[ref54] Zhang W. Zheng R. (2004). Subjective well-being in college students. Chin. Ment. Health J. 1, 61–62. doi: 10.3321/j.issn:1000-6729.2004.01.022

